# Supplementary material for: Development and validation of real-time recombinase polymerase amplification-based assays for detecting HPV16 and HPV18 DNA
Source: Microbiol Spectr. 2023 Oct 3;11(6):e01207-23. doi: 10.1128/spectrum.01207-23 (PMC10714791; doi:10.1128/spectrum.01207-23)
Supplement: Supplemental file 1 — Fig. S1 and Tables S1 to S4. [file spectrum.01207-23-s0001.docx]

**Table S1.** **Patient characteristics.** Unknown, patient information not available. HSIL, high-grade squamous intraepithelial lesions; LSIL, low-grade squamous intraepithelial lesions; NILM, negative for intraepithelial lesion or malignancy.

| **Patient characteristics** | | **No. of patients** | **% of patients (out of 43)** |
| --- | --- | --- | --- |
| **Age, years** |  |  |  |
|  | Median | 43 |  |
|  | Range | 26-68 |  |
| **Category** |  |  |  |
|  | Cervical cancer | 18 | 41.86% |
|  | HSIL | 17 | 39.54% |
|  | LSIL | 4 | 9.30% |
|  | NILM | 4 | 9.30% |
| **HPV infection genotype** | |  |  |
|  | HPV16 | 30 | 69.77% |
|  | HPV18 | 9 | 20.93% |
|  | Co-infection with HPV16/18 | 4 | 9.30% |
| **Place of residence** | |  |  |
|  | City | 24 | 55.81% |
|  | Rural | 15 | 34.88% |
|  | Unknown | 4 | 9.30% |
| **Number of children** | |  |  |
|  | None | 8 | 18.60% |
|  | One | 27 | 62.79% |
|  | More than one | 7 | 16.28% |
|  | Unknown | 1 | 2.33% |
| **Menopause** |  |  |  |
|  | Menopausal | 14 | 32.56% |
|  | Pre-menopausal | 28 | 65.12% |
|  | Unknown | 1 | 2.33% |
| **Age at the start of sexual activity (years)** | |  |  |
|  | Median | 22 |  |
|  | <20 | 6 | 13.95% |
|  | ≧20 | 31 | 72.09% |
|  | Unknown | 6 | 13.95% |
| **Smoking history** | |  |  |
|  | Currently smoker | 2 | 4.65% |
|  | Former smoker | 1 | 2.33% |
|  | Never smoked | 38 | 88.37% |
|  | Unknown | 2 | 4.65% |
| **Alcohol history** | |  |  |
|  | Currently drinker | 2 | 4.65% |
|  | Former drinker | 2 | 4.65% |
|  | Never drank | 37 | 86.05% |
|  | Unknown | 2 | 4.65% |
| **HPV test history** | |  |  |
|  | First test | 32 | 74.42% |
|  | Tested in the past 3 years | 10 | 23.26% |
|  | Unknown | 1 | 2.33% |
| **HPV vaccination** | |  |  |
|  | Vaccinated | 0 | 0.00% |
|  | Unvaccinated | 38 | 88.37% |
|  | Unknown | 5 | 11.63% |

**Table S2.** **Detection of HPV16 gene segments in clinical samples with three RPA-exo assays relative to real-time PCR (qPCR).** TCT, ThinPrep cytological test samples; +: positive; −: negative; gray boxes: inconsistent with qPCR results; β-globin-negative: test invalid (DNA degraded). All samples were tested in triplicate, and each test had a negative control and a positive control. + or – without parentheses indicate consistent results between all replicates. The numbers in parentheses indicate the number of positive results out of the total number of replicates.

| **Sample ID** | **Biopsy** | | | | **TCT** | | | |
| --- | --- | --- | --- | --- | --- | --- | --- | --- |
|  | **qPCR Ct** | **L1 RPA-exo** | **E7 RPA-exo** | **Dual RPA-exo** | **qPCR Ct** | **L1 RPA-exo** | **E7 RPA-exo** | **Dual RPA-exo** |
| 10 | 24.1 | - | + | + | 21.3 | + | + | + |
| 16 | 16.8 | + | + | + | 23.3 | + | + | + |
| 26 | 23.1 | + | + | + | 26.3 | + | + | + |
| 27 | 18.6 | + | + | + | 19.3 | + | + | + |
| 41 | 30.0 | + | + | + | 28.7 | + | + | + |
| 42 | 30.5 | + | + | + | 22.0 | + | + | + |
| 50 | 32.1 | + | + | + | 24.7 | + | + | + |
| 53 | - | - | - | - | 26.5 | + | + | + |
| 55 | 23.9 | + | + | + | 12.6 | + | + | + |
| 56 | 20.7 | + | + | + | 23.6 | + | + | + |
| 65 | 27.4 | + | + | + | 18.4 | + | + | + |
| 79 | - | - | - (1/3) | - | 35.4 | - | + | - |
| 81 | 29.8 | + | + | + | 26.9 | + | + | + |
| 82 | 37.1 | - | - (1/6) | - | 27.0 | + | + | + |
| 83 | 33.7 | + | + (2/3) | + (4/6) | 24.6 | + | + | + |
| 96 | 23.0 | + | + | + | 24.3 | + | + | + |
| 105 | - | - (1/3) | - | - | - | - | - | - |
| 106 | β-globin-negative | | | | 34.3 | + | - (1/6) | + (2/3) |
| 110 | 37.5 | - | - | - | 29.3 | + | + | + |
| 112 | 23.6 | + | + | + | 29.1 | + | + | + |
| 114 | 39.1 | - | - | - | 26.6 | + | + | + |
| 118 | 26.5 | + | + | + | 21.2 | + | + | + (2/3) |
| 121 | NO SAMPLE | | | | 36.5 | - (1/6) | - | - |
| 124 | - | - | - | - | - | - | - | - |
| 130 | NO SAMPLE | | | | 18.7 | + | + | + |
| 137 | NO SAMPLE | | | | 23.9 | + | + | + |
| 142 | 25.3 | + | + | + | 24.6 | + | + | + |
| 144 | NO SAMPLE | | | | 19.6 | + | + | + |
| 149 | 19.7 | + | + | + | - | - | - | - |
| 152 | NO SAMPLE | | | | 24.6 | + | + | + |
| 153 | 31.9 | + (2/3) | - | + | 21.6 | + | - | + |
| 165 | 16.9 | + | + | + | 17.0 | + | + | + |
| 167 | - | - | - | - | 31.9 | + | - | + |
| 175 | 22.9 | + | + | + | 23.2 | + | + | + |

**Table S3.** **Detection of HPV18 gene segments in clinical samples with three RPA-exo assays relative to real-time PCR (qPCR).** TCT, ThinPrep cytological test samples; +: positive; −: negative; gray boxes: inconsistent with qPCR results. All samples were tested in triplicate, and each experiment had a negative control and a positive control. + or − indicate consistent results between all replicates. The numbers in parentheses indicate the number of positive results out of the total number of replicates.

| **Sample ID** | **Biopsy** | | | | **TCT** | | | |
| --- | --- | --- | --- | --- | --- | --- | --- | --- |
|  | **qPCR Ct** | **L1 RPA-exo** | **E7 RPA-exo** | **Dual RPA-exo** | **qPCR Ct** | **L1 RPA-exo** | **E7 RPA-exo** | **Dual RPA-exo** |
| 32 | 27.6 | + | + | + | 22.7 | + | + | + |
| 57 | - | - | - | - | 36.7 | - | - | - |
| 67 | - | - | - (1/6) | - | 32.1 | + | - (1/6) | - (1/3) |
| 68 | 28.0 | + | + | + (2/3) | 34.4 | + | + | + (2/3) |
| 79 | - | - | - | - (2/6) | - | - | - | - |
| 81 | - | - | - | - | - | - | - | - |
| 94 | NO SAMPLE | | | | 23.9 | + | + | + |
| 102 | NO SAMPLE | | | | 33.7 | + | + | + |
| 103 | 20.6 | + | +(2/3) | + | 28.2 | + | + | + |
| 116 | 36.7 | - (1/3) | - (1/6) | - | NO SAMPLE | | | |
| 144 | NO SAMPLE | | | | - | - | - | - |
| 151 | - | - | - | - | - | - | - | - |
| 175 | - | - | - | - | - | - | - | - |

**Table S4.** **RPA-exo detection of ThinPrep cytological test DNA extracted via other methods.** +: positive; −: negative. All samples were tested in triplicate, and each test had a negative control and a positive control. + or – without parentheses indicate consistent results between all replicates. gray boxes: inconsistent with phenol-chloroform extracted DNA results. The numbers in parentheses indicate the number of positive results out of the total number of replicates.

| **HPV** | **Sample ID** | **L1 RPA- exo** | **E7 RPA-exo** | **Dual RPA-exo** |
| --- | --- | --- | --- | --- |
| HPV 16 | 16 | + | + | 16+ 18- |
|  | 27 | + | + | 16+ 18- |
|  | 41 | + | + | 16+ 18- |
|  | 42 | + | + | 16+ 18- |
|  | 50 | + | + | 16+ 18- |
|  | 53 | + | + | 16+ 18- |
|  | 55 | + | + | 16+ 18- |
|  | 56 | + | + | 16+ 18- |
|  | 65 | + | + | 16+ 18- |
|  | 110 | + | + | 16+ 18- |
|  | 130 | + | + | 16+ 18- |
|  | 149 | - | - | 16- 18- |
| HPV18 | 32 | + | + | 16- 18+ |
|  | 57 | - | - | 16- 18- |
|  | 67 | + | + | 16- 18+ |
|  | 68 | + | + | 16- 18+ |
|  | 102 | + | +(4+ 1-) | 16- 18+ |
|  | 103 | + | + | 16- 18+ |
| Co-infection | 81 | 16+ 18- | 16+ 18- | 16+ 18- |
|  | 94 | 16- 18+ | 16- 18+ | 16- 18+ |
| Crude DNA extraction | 27 | + | + | 16+ 18- |
|  | 56 | + | + | 16- 18- |
|  | 65 | + | + | 16- 18- |


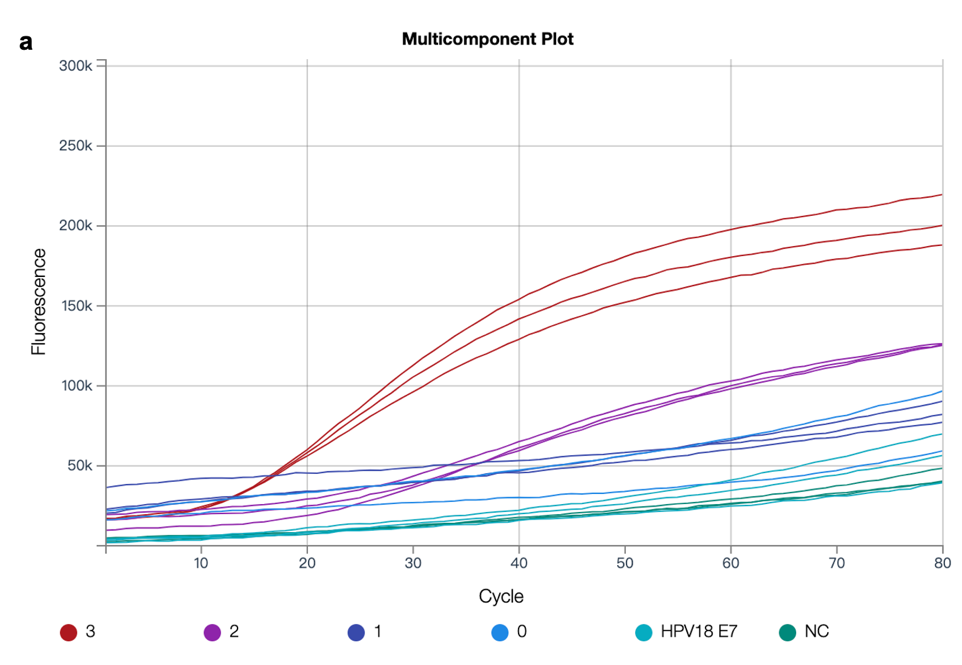

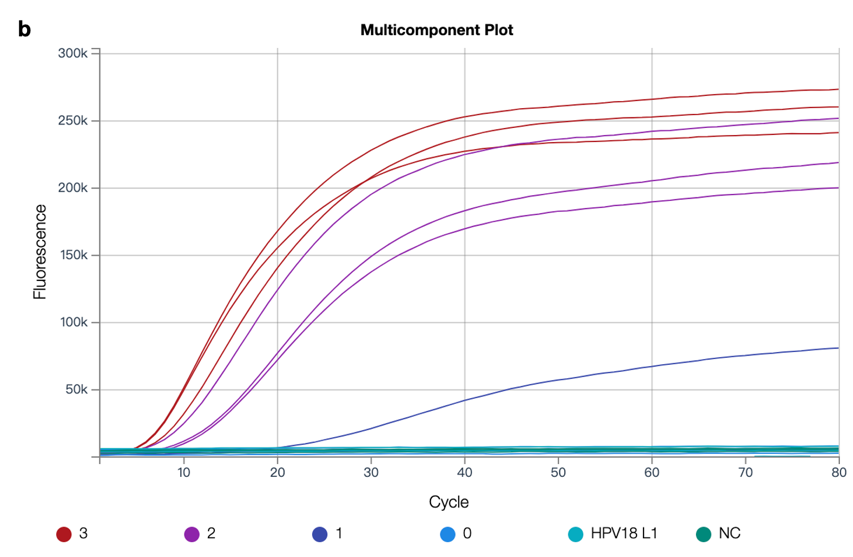


**Figure** **S1.** **Background fluorescence readings for the E7 and L1 RPA-exo assays.** Serial 10-fold dilutions of HPV16 E7 and L1 DNA plasmids (10^3^-10^0^ copies) were subjected to RPA-exo assays. 3, 2, 1, 0 represent the log_10_ of viral DNA copies per reaction. NC is the negative control, nuclease-free water in place of the DNA. Each cycle corresponds to 15 s. (a) HPV16 E7 RPA-exo assay, the fluorescence value of the NC increased over time. HPV18 E7 indicates that the HPV18 E7 plasmid was added. (b) HPV16 L1 RPA-exo assay, the fluorescence value of the NC did not increase over time. HPV18 L1 indicates that the HPV18 L1 plasmid was added.
